# Missed opportunities of feedback for emergency ambulance staff: a mixed-methods diary study

**DOI:** 10.29045/14784726.2025.6.10.1.27

**Published:** 2025-06-01

**Authors:** Caitlin Wilson, Luke Budworth, Gillian Janes, Rebecca Lawton, Jonathan Benn

**Affiliations:** University of Leeds; Yorkshire Ambulance Service Research Institute; Yorkshire Quality and Safety Research Group ORCID iD: https://orcid.org/0000-0002-9854-4289; Yorkshire Quality and Safety Research Group; NIHR Yorkshire & Humber Patient Safety Research Collaboration ORCID iD: https://orcid.org/0000-0002-2905-6011; Manchester Metropolitan University; Anglia Ruskin University ORCID iD: https://orcid.org/0000-0002-1609-5898; University of Leeds; Yorkshire Quality and Safety Research Group; NIHR Yorkshire & Humber Patient Safety Research Collaboration ORCID iD: https://orcid.org/0000-0002-5832-402X; University of Leeds; Yorkshire Quality and Safety Research Group; NIHR Yorkshire & Humber Patient Safety Research Collaboration ORCID iD: https://orcid.org/0000-0001-5919-9905

**Keywords:** ambulances, feedback, surveys and questionnaires

## Abstract

**Introduction:**

Providing feedback to emergency ambulance staff on performance or patient outcomes may improve care quality and professional development. Current feedback provision in ambulance services is limited and staff desire more feedback; however, we do not know what feedback would be most useful. This study aimed to determine the quality of feedback received by emergency ambulance staff, describe self-directed learning activities performed after receiving feedback (e.g. ‘reflected on what exactly I did right/wrong’) and identify situations where ambulance staff desired enhanced feedback.

**Methods:**

An observational mixed-methods study was used. Emergency ambulance staff delivering face-to-face patient care in the United Kingdom’s National Health Service completed a baseline survey and diary entries between March and August 2022. Diary entries were event contingent and were collected when a participant identified that they had received feedback or desired feedback but had not received it. Free-text qualitative responses were categorised using content analysis before being included in the quantitative analyses. Quantitative data were analysed using descriptive and inferential statistics.

**Results:**

Baseline surveys were completed by 299 participants; 100 participants submitted 374 feedback-desired diary entries and 105 participants submitted 538 feedback-received diary entries. Ambulance staff expressed a statistically significant preference for patient-outcome feedback (77.8% [95% CI 74.0, 82.1]), provided by non-ambulance healthcare professionals (70.7% [66.2, 75.3]) and delivered electronically (54.0% [48.9, 59.4]). Feedback was particularly desired for cases involving neurological (17.1%) and cardiovascular (16.6%) conditions and non-conveyed patients (11.5%). Self-directed learning activities post feedback included reflection (61.5%), considering alignment with own judgement (41.1%) and discussions with colleagues (37.0%).

**Conclusion:**

The study identifies critical gaps in current feedback practices within ambulance services and provides directions for feedback designs that would enhance existing systems and approaches. Training programmes should educate ambulance staff on effective feedback utilisation and management of both positive and negative feedback. Cultivating a supportive feedback culture within ambulance services is crucial for fostering continuous professional growth and improving patient care outcomes.

## Introduction

Emergency ambulance staff face high levels of work-related stress and burnout and increased likelihood that they will form intentions to leave their jobs, as identified by the NHS staff survey (NHS England, 2023). Contributing to this stress are the complexities, uncertainties and extreme stressors inherent in the ambulance service work environment (Fisher et al., 2015; Lawn et al., 2020). Despite this, ambulance staff work autonomously, making critical decisions to treat patients at home, which can reduce unnecessary hospital attendance and alleviate emergency department (ED) demand (Blodgett et al., 2021; Paulin et al., 2021).

Receiving feedback on patient outcomes and personal performance has been suggested as a means to improve job support for ambulance staff, potentially enhancing staff well-being, job satisfaction and patient care (Eaton-Williams et al., 2020; Wilson et al., 2022). Clinical performance feedback has shown positive effects on the quality of care and professional development across various healthcare settings, including ambulance services (Ivers et al., 2012; Wilson et al., 2023b).

Performance feedback generally centres on an individual’s adherence to clinical protocols and skill proficiency, offering specific, actionable insights for skill development and professional growth (Brown et al., 2019; Hysong et al., 2017). In contrast, patient outcome feedback or follow-up involves clinicians receiving information about patients’ diagnoses, treatments and clinical progress after initial care (Cifra et al., 2021). While performance feedback offers concrete guidance for improving daily practices, outcome feedback fosters reflection and self-evaluation, enhancing diagnostic insight, promoting behaviour change and improving the calibration between diagnostic accuracy and clinician confidence (Fernandez Branson et al., 2021).

However, feedback provision in ambulance services is challenging due to factors such as a mobile workforce, disconnected digital technology and data-sharing governance issues (Eaton-Williams et al., 2020; Porter et al., 2020). Currently, feedback to ambulance staff is often provided through formal initiatives, such as performance appraisals, ‘post-box’ schemes for patient outcome feedback and patient-experience feedback via thank-you letters or the Friends and Family Test (Wilson et al., 2022, 2023a). When formal feedback mechanisms are lacking, ambulance staff may seek informal feedback from ED staff, although this is limited by patient confidentiality, information quality and logistical barriers (Eaton-Williams et al., 2020; Morrison et al., 2017).

The broader feedback literature suggests that a deeper understanding of feedback context, format and mechanisms is required to improve feedback effectiveness (Hysong et al., 2017). An example of this is Clinical Performance Feedback Intervention Theory (Brown et al., 2019), which has good face validity in the pre-hospital setting (Wilson et al., 2022) and offers 42 hypotheses of when feedback is more effective (e.g. when feeding back to staff with positive beliefs about feedback).

Alongside the literature on feedback interventions and effectiveness, a separate stream of literature has focused on feedback-seeking behaviour, with seminal work by Ashford and Cummings (1983) emphasising that employees may actively seek feedback rather than passively wait for feedback. A systematic review of pre-hospital feedback initiatives (Wilson et al., 2023b) identified only one study where ambulance staff had to actively request feedback (Stella et al., 2010). However, a practice review of 40 feedback initiatives in UK ambulance services identified that nearly two thirds required active feedback-seeking (Wilson et al., 2023a), emphasising the relevance of both feedback intervention and feedback-seeking literature to pre-hospital feedback initiatives.

Qualitative research indicates that ambulance staff desire more comprehensive and timely feedback, particularly regarding patient outcomes (Eaton-Williams et al., 2020; Morrison et al., 2017; Wilson et al., 2022). However, a deeper understanding is needed of how ambulance staff perceive the quality of the feedback that they receive, how they respond to current feedback and what feedback they desire to inform the development of feedback mechanisms that enhance their clinical practice and personal well-being.

This study aimed to answer the following research questions:

- What is the self-reported quality of feedback received by emergency ambulance staff?
- How do emergency ambulance staff engage with the feedback they receive and what do they do with it?
- In which situations do emergency ambulance staff desire increased feedback?

## Methods

### Study design

This sub-study is part of a broader observational mixed-methods study, which consisted of a baseline survey, diary entries (received and desired feedback) and a follow-up survey. Results of the baseline survey and ‘feedback-received diary entries’ are reported separately (Wilson et al., 2024). The present sub-study relied upon analysis of data collected during the feedback-received and feedback-desired diary entries and from the final follow-up survey. Collecting diary entries in real time is known to reduce recall bias by collecting data at the level of feedback events and therefore not relying on generalised reflections on feedback provision over a period of time. At the same time this enables analysis of within- and between-person variability (Bolger et al., 2003). Diary entries were event contingent and were collected when a participant identified that they had either received feedback (‘feedback-received diary entry’) or had an unfulfilled desire for feedback (‘feedback-desired diary entry’).

Ethical approval was granted from the University of Leeds ethics committee (PSYC-406 04/01/2022) and the Health Research Authority (ID: 295645).

STROBE (Elm et al., 2007) recommendations were followed.

### Setting and selection of participants

Eligible participants were registered ambulance clinicians (i.e. paramedics) and non-registered staff (e.g. emergency medical technicians) delivering face-to-face patient care and employed by an NHS ambulance trust in the UK.

An opportunistic sample was recruited via social media and the internal communications of relevant organisations. Informed consent was obtained in the baseline survey after providing study information. Access to the baseline survey was via an anonymous link, with individual diary study links issued to participants who provided their email address in their survey response. Participants completing all study elements were enrolled in a prize draw for three £50 vouchers to aid recruitment and reduce drop-out.

### Study size

A power calculation was performed for the study elements reported elsewhere (Wilson et al., 2024), suggesting that 325 participants were required. No separate power calculation was performed for the exploratory analyses of the feedback-desired diary entries and follow-up survey reported here.

### Data collection

Data were collected using Qualtrics (Qualtrics) between March and August 2022. The diary study measure and follow-up survey were developed for this study (Supplementary 1). These were piloted with three ambulance staff and were refined based on their feedback.

Immediately after completing the baseline survey, participants were sent a link to access their diary, which remained open until the end of the data collection period. When logging a feedback-desired event, participants were asked a series of multiple-choice and structured-response questions informed by Clinical Performance Feedback Intervention Theory (Brown et al., 2019), including, for example, ‘Who would you like this feedback to be provided by?’ and ‘How would you like this to be provided?’.

Similarly, participants logging feedback-received events were asked who the feedback had been provided by and how. For feedback-received events, participants were also asked to indicate which self-directed learning activity they had undertaken as a result of the feedback using an established list; for example, ‘read professional books/journals’ or ‘reflected on what exactly I did right/wrong’, drawing upon a previous diary study on feedback across the education, healthcare and profit sector by Mulder (2013). When a participant reached the required number of 15 total diary entries, they were sent a follow-up survey prompting them to reflect on their diary study experience.

The feedback quality measures were included in the feedback-received diary entries (‘usefulness’) and follow-up survey (‘quality’). Participants were asked to respond on a Likert-type scale ranging from 1 to 7 as to how they would rate the usefulness (‘not useful at all’ to ‘extremely useful’) and overall quality (‘extremely poor quality’ to ‘extremely good quality’) of the feedback they received.

### Data analysis

The mixed-methods nature of this study follows the approach defined by Creswell and Plano Clark (2017) as ‘triangulation design: validation quantitative data model’. The primary emphasis of data collection in this study was quantitative survey data, which provided core numerical insights. To complement and validate these findings, qualitative free-text survey items were included. This inclusion allowed participants to highlight and describe categories that had not been previously identified by the research team through existing literature and piloting.

Free-text qualitative responses were categorised in NVivo (Version 12 Plus, QSR International) using content analysis before being included in the quantitative analyses. This was undertaken by an early-career paramedic researcher (CW), with input from the wider research team of senior health services researchers (GJ, RL, JB) with expertise in patient safety research, implementation and behavioural science.

Quantitative data were analysed using descriptive and inferential statistics in R (Version 4.1.3, R Core Team). Descriptive statistics were presented as frequency and percentage for categorical variables and median and interquartile range (IQR) for skewed continuous variables. Inferential statistics consisted of performing a chi-square test and Fisher’s exact test, with p <0.05 indicating statistical significance, and calculating simultaneous confidence intervals (CIs) for the probabilities of a multinomial distribution using the R package ‘MultinomialCI’, following the method proposed by Sison and Glaz (1995).

Figures were developed using the R package ‘ggplot’ and combined using ‘patchwork’.

## Results

### Characteristics of study participants

**Figure 1:**
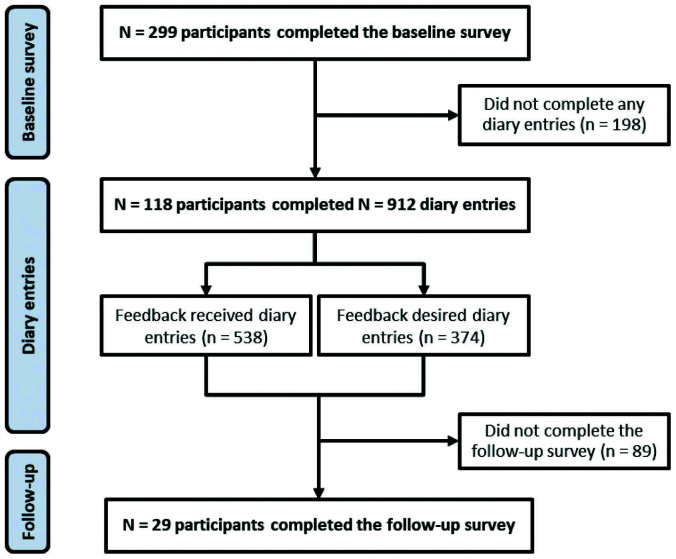
Figure 1. Flow diagram depicting the number of participants throughout the research study.

The baseline survey was completed by 299 participants, representing 13 of the 14 UK ambulance trusts (range 4‒88, median 19 participants per trust). Of these, 100 completed 374 feedback-desired diary entries (range 1‒15, median 3.5), 105 completed feedback-received diary entries (range 1‒16, median 4) and 29 completed follow-up surveys. Figure 1 depicts participant flow.

Table 1 summarises participants’ baseline characteristics. Ethnicity was collapsed into a binary variable (white n = 290; minoritised ethnic group n = 8) to avoid identifying participants. Inferential statistics did not indicate that participants’ characteristics significantly differed between the baseline survey and diary entry stages. Comparison with national data for UK ambulance service staff (NHS Digital, 2022a) using chi-square tests at 0.05 significance level indicated that our study sample was representative in terms of ethnicity (p = 0.771), sex (p = 0.124) and age (p = 0.886).

**Table 1.** Characteristics of study participants.

	Baseline survey	Diary: feedback received	Diary: feedback desired
Number of participants, n	299	105	100
Role, n (%)			
Emergency medical technician	59 (19.7)	16 (15.2)	16 (16.0)
Paramedic	239 (79.9)	89 (84.8)	84 (84.0)
Age in years, median (IQR)	36 (29.0–45.0)	38 (30.5–45.0)	38 (31.3–46.8)
Sex, n (%)			
Female	120 (40.1)	39 (37.1)	33 (33.0)
Male	177 (59.2)	66 (62.9)	67 (67.0)
Not stated	2 (0.7)	0 (0)	0 (0)
Ethnicity, n (%)			
Minoritised ethnic group	8 (2.7)	2 (1.9)	2 (2.0)
White	290 (97.0)	103 (98.1)	98 (98.0)
Not stated	1 (0.3)	0 (0)	0 (0)
Years of work experience, median (IQR)	7 (3.7–13.4)	9 (4.5–14.4)	8.9 (4.5–14.5)
Presence of formal feedback initiative, n (%)			
Yes	68 (22.7)	26 (24.8)	23 (23.0)
No	231 (77.3)	79 (75.2)	77 (77.0)

### Quality of feedback received

Participants indicated that instances of received feedback answered their questions 46.7% of the time (n = 250), while their questions remained unanswered 3.2% of the time (n = 17). For the remainder of feedback-received events (n = 271, 50.4%), participants responded that this survey question was not applicable, suggesting that the desired purpose of feedback may not always be related to a particular question that staff have.

The median rating of the usefulness of feedback received following diary entries where participants received feedback was 6 (IQR 5‒7), and the median quality was 4 (IQR 4‒6), indicating that feedback was found to be very useful but only of adequate quality.

### Self-directed learning activities

Table 2 provides a descriptive summary of participants’ self-directed learning activities after receiving feedback. They most often reported having reflected on what exactly they did right or wrong (n = 331, 61.5%). To a lesser extent, they thought about whether the feedback matched their own judgement (n = 221, 41.1%), discussed the feedback with their line manager or colleagues (n = 199, 37.0%) and passed their new knowledge or skills onto others (n = 144, 26.8%).

**Table 2.** Descriptive summary of self-directed learning activities.

Self-directed learning activity	n (%)
Reflected on what exactly I did right/wrong	331 (61.5)
Thought about whether the feedback regarding my work matches my own judgement	221 (41.1)
Discussed the feedback with my line manager/colleagues/others	199 (37.0)
Passed on my knowledge/skills to others	144 (26.8)
Observed how others work	87 (16.2)
Read professional books/journals	62 (11.5)
Searched for help/solutions on the internet	46 (8.6)
Changed my clinical practice	43 (8.0)
Asked my supervisor or colleagues for advice	34 (6.3)
Took part in training opportunities	13 (2.4)

### Opportunities for enhanced feedback

**Figure 2:**
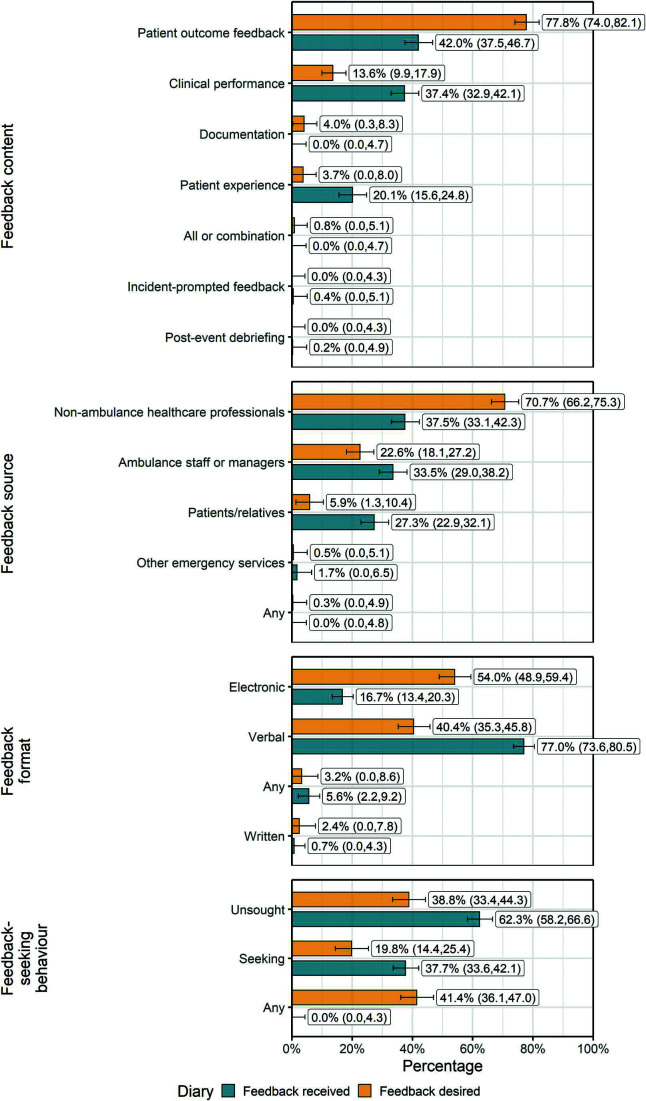

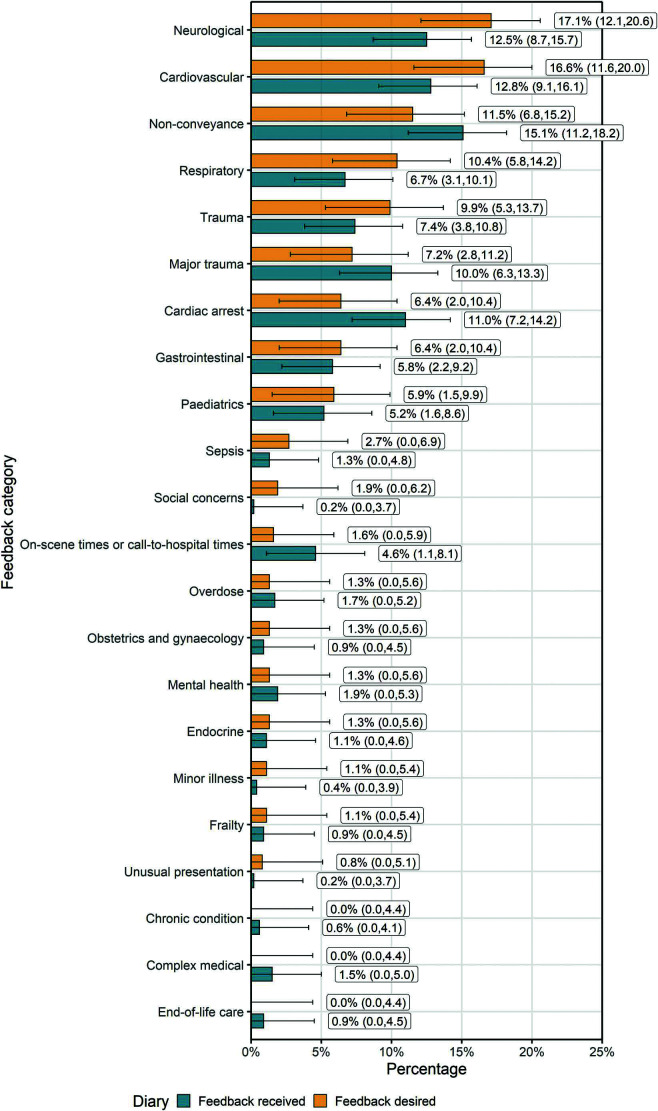
Figure 2. Characteristics and comparison of feedback-desired and feedback-received diary entries.

The findings displayed in Figure 2 highlight significant discrepancies between desired and received feedback among emergency ambulance staff.

In terms of feedback content, patient-outcome feedback was overwhelmingly desired (n = 291, 77.8% [95% CI 74.0, 82.1]), while actual receipt of this was considerably lower (n = 226, 42.0% [37.5, 46.7]) (p <0.001). Feedback on clinical performance (n = 51, 13.6% [9.9, 17.9]) and patient experience (n = 14, 3.7% [0.0, 8.0]) were less desired but were more frequently received (n = 201, 37.4% [32.9, 42.1] and n = 108, 20.1% [15.6, 24.8], respectively).

Participants indicated most frequently that they wanted feedback from healthcare professionals outside the ambulance service (n = 266, 71.1% [66.2, 75.3]), yet actual receipt from this source was notably lower (n = 202, 37.5% [33.1, 42.4]) (p <0.001).

The preferred format for feedback was electronic (n = 202, 54.0% [48.9, 59.4]) rather than verbal (n = 151, 40.4% [35.3, 45.8]), yet the actual receipt of electronic feedback was much lower (n = 90, 16.7% [13.4, 20.3]) compared to verbal feedback (n = 414, 77.0% [73.6, 80.5]) (p <0.001).

Fewer than one in five participants preferred pull feedback, where they would need to actively seek it themselves (n = 74, 19.8% [14.4, 25.4]). Most participants desired feedback that was provided without seeking (n = 145, 38.8% [33.4, 44.3]), meaning that feedback would be provided automatically or instigated externally to the recipient, or that feedback was provided by either method (n = 155, 41.4% [36.1, 47.0]).

There were 22 categories of patient conditions that participants desired feedback for, with most diary entries indicating more than one category. Nine of these categories were prompted within the survey, with a further 13 identified inductively from survey responses. Participants most frequently desired feedback on patients presenting with neurological (n = 64, 17.1% [12.1, 20.6]) or cardiovascular (n = 62, 16.6% [11.6, 20.0]) symptoms or those who were not conveyed to hospital (n = 32, 11.5% [6.8, 15.2]) (Figure 2). A chi-square test was not performed for this variable due to the high number of different categories and resulting low power.

## Discussion

Our study makes a novel contribution to the literature by characterising the self-reported quality of pre-hospital feedback, describing how ambulance staff engage with feedback and outlining situations in which emergency ambulance staff desire increased feedback. Ambulance staff perceived feedback to be very useful but only of adequate quality. Receiving feedback allowed ambulance staff to reflect on what they had done right or wrong.

Although ambulance staff provide care for an undifferentiated population of patients, they most desire feedback for patients with neurological (17.1%) and cardiovascular conditions (16.6%), as well as those who are not conveyed to hospital (11.5%). Although the desire for feedback on non-conveyed patients is unsurprising given the high variability in conveyance rates (O’Cathain et al., 2018) and the need to improve patient safety in this area (Ebben et al., 2017), it highlights a clear need for development, as our review of practice found no feedback initiatives spanning the boundary between ambulance services and community services or primary care physicians in the UK (Wilson et al., 2023a).

Compared to other feedback types, patient-experience feedback was desired less, which differs from the shift in healthcare generally towards more patient-centred care and many NHS trusts using this type of feedback as key performance indicators (NIHR Dissemination Centre, 2019). Our participants predominantly desired patient-outcome feedback (77.8%), delivered by non-ambulance healthcare professionals (70.7%) and without requiring active feedback-seeking (38.8%). This may be due to the perception that ambulance colleagues lack access to the type of detailed, long-term patient-outcome information that is typically available to hospital clinicians. Furthermore, participants’ desire for non-active feedback highlights a preference for easily available feedback rather than the burden of active seeking, underscoring the value of improved interdisciplinary information sharing to support effective feedback.

The desire for either automated or externally generated feedback could also be a call towards feedback provision being systematised and embedded within the system. However, there may be advantages to encouraging more active feedback-seeking behaviour, as it allows recipients to target feedback on particular cases or skills they wish to improve (Crommelinck & Anseel, 2013). Ashford and Cummings (1983) suggest that acceptance of feedback and the desire to respond in line with the feedback are different when feedback is actively sought from when it is passively received. Actively seeking feedback can foster a more tailored, meaningful exchange that better addresses the individual’s immediate learning needs, enhancing the feedback’s practical value and impact.

Our participants’ desire for unsought feedback delivered electronically (54.0%) may also hint at the current service data infrastructure not adequately meeting staff needs. This underscores the importance of developing systems that provide systematic, routine, relevant and timely feedback to minimise unanswered questions. Such an approach aligns with CP-FIT (Brown et al., 2019), which highlights the value of automation in data collection to reduce manual effort, ensuring that feedback is timely and actionable.

The finding that, for more than half of the feedback-received events, participants indicated that answering their question was not applicable, suggests that feedback may serve broader functions beyond addressing specific, pre-existing questions or uncertainties. This highlights the potential for feedback to play a more proactive role, such as fostering general professional development, affirming good practice or prompting reflective learning. It may also indicate that staff value feedback as a mechanism for ongoing improvement rather than solely as a tool for resolving immediate issues (Ivers et al., 2012). Developing feedback systems that account for these broader purposes, alongside addressing specific information needs, could enhance their relevance and utility for ambulance staff.

Consistent with proposals within the broader literature on feedback intervention and feedback-seeking behaviour research (Anseel et al., 2018; Denisi & Sockbeson, 2018), integration and advances in both unsought and actively sought feedback are needed to gain a deeper understanding of feedback and its effects within ambulance services.

### Implications for research and practice

This study highlights several critical areas for future research and practical improvements in the feedback mechanisms for ambulance services. More research is needed to delve deeper into the self-directed learning activities ambulance staff engage in after receiving feedback. Understanding these activities can help develop targeted educational and training programmes that complement formal feedback mechanisms. Additionally, exploring the relationship between patient-experience feedback and patient safety in pre-hospital emergency settings – a relationship well established in hospital contexts but less so in ambulance services – could yield important insight. Investigating whether and how patient-outcome feedback demonstrates effective cross-boundary working or can be leveraged to improve clinical decision making and patient safety could address existing gaps and advance feedback practices.

Ambulance services should invest in developing robust feedback systems that facilitate timely and relevant feedback. This includes integrating digital platforms that enable seamless sharing of patient-outcome information across different healthcare settings. Given the desire for feedback from non-ambulance healthcare professionals, establishing stronger interdepartmental and interdisciplinary communication channels is crucial. This can be achieved through formalised feedback loops involving hospitals, primary care and community services.

### Strengths and limitations

This was the first study to explore the feedback desired by ambulance staff and to assess the quality of the feedback received. This study was limited by the high drop-out rate (n = 299 participants at baseline, n = 100 logging diary entries, n = 29 at follow-up), though this is typical of diary studies generally (Ohly et al., 2010). Nevertheless, comparison with national data for UK ambulance services (NHS Digital, 2022b) indicated that our study sample was representative of UK ambulance staff. There are differences in the way ambulance services in the four nations of the UK are commissioned and operated, but due to low samples, this study was not able to explore differences with regard to desired feedback.

Despite data collection taking place during the early post-pandemic period, when the backlog of health needs was emerging, the large number of NHS staff that participated and the feedback events that were reported indicate an appetite for feedback research from ambulance staff, and this enabled us to confidently address the study objectives reported here. However, this study was unable to recruit to target. Challenges related to the demanding schedules and limited availability for research participation of the target NHS staff group, combined with reliance on voluntary participation, are likely to have contributed to the relatively low response rate. The use of diaries collected via an online survey, while novel and flexible, may also have influenced participation. While the diary-keeping process may have encouraged more reflective responses in the follow-up survey, the time commitment required could have deterred some participants. Future research should explore alternative recruitment strategies and greater incentives or should further reduce survey length to enhance participation rates within this professional context.

## Conclusion

This study identifies critical gaps in current feedback practices within ambulance services and provides directions for feedback designs that would enhance existing systems and approaches. Our findings indicate that ambulance staff perceived feedback to be very useful but only of adequate quality. Receiving feedback facilitated reflection on what was done right or wrong. Feedback was particularly desired regarding patients with neurological and cardiovascular conditions, as well as those not conveyed to hospital. Future research should focus on measuring feedback outcomes (including self-directed learning activities) and integrating seamless, interdisciplinary digital systems to provide feedback for emergency ambulance staff. By addressing these needs, ambulance services can foster a supportive feedback culture, enhancing both job satisfaction and patient outcomes.

## Acknowledgements

The authors would like to thank the study participants for taking the time to complete the survey and to log diary entries, as well as the research departments of participating ambulance trusts for their support with advertisement and recruitment. Thank you to Professor Helen Snooks, University of Swansea, and Professor Graham Law, University of Lincoln, for peer-reviewing the study protocol.

## Authors contributions

CW conceived the study, developed the study protocol, obtained relevant ethics and governance approvals, collected the data, analysed the data and drafted the manuscript under supervision from GJ, RL and JB. LB provided guidance on the sample size calculation and data analysis. CW drafted the article, and all authors contributed substantially to its revision and approved the final version. CW acts as the guarantor for this article.

## Conflict of interest

CW is an associate editor for the *British Paramedic Journal*.

## Ethics

The study was carried out in accordance with the UK Policy Framework for Health and Social Care Research (Health Research Authority, 2017) and was approved by the Health Research Authority (IRAS project ID 295645) and the University of Leeds ethics committee (PSYC-406 04/01/2022). Informed consent was obtained in the baseline survey after providing participants with study information. The datasets generated and analysed during the current study are not publicly available, as sharing the raw data would violate the agreement to which participants consented; however, the datasets are available from the corresponding author on reasonable request.

## Funding

This research was funded by the National Institute for Health Research Yorkshire and Humber Patient Safety Translational Research Centre (NIHR Yorkshire and Humber PSTRC). The views expressed are those of the authors and not necessarily those of the NIHR or the Department of Health and Social Care. The funders had no role in study design, data collection and analysis, the decision to publish or the preparation of the manuscript.
